# At*MND1 *is required for homologous pairing during meiosis in *Arabidopsis*

**DOI:** 10.1186/1471-2199-7-24

**Published:** 2006-07-27

**Authors:** Aneesh P Panoli, Maruthachalam Ravi, Jose Sebastian, Bindu Nishal, Thamalampudi V Reddy, Mohan PA Marimuthu, Veeraputhiran Subbiah, Virupapuram Vijaybhaskar, Imran Siddiqi

**Affiliations:** 1 Centre for Cellular and Molecular Biology, Uppal Road, Hyderabad – 500007, India; 2 Present address: Biotechnology Division, Institute of Himalayan Bioresource Technology, Palampur – 176061, Himachal Pradesh, India

## Abstract

**Background:**

Pairing of homologous chromosomes at meiosis is an important requirement for recombination and balanced chromosome segregation among the products of meiotic division. Recombination is initiated by double strand breaks (DSBs) made by Spo11 followed by interaction of DSB sites with a homologous chromosome. This interaction requires the strand exchange proteins Rad51 and Dmc1 that bind to single stranded regions created by resection of ends at the site of DSBs and promote interactions with uncut DNA on the homologous partner. Recombination is also considered to be dependent on factors that stabilize interactions between homologous chromosomes. In budding yeast Hop2 and Mnd1 act as a complex to promote homologous pairing and recombination in conjunction with Rad51 and Dmc1.

**Results:**

We have analyzed the function of the *Arabidopsis *orthologue of the budding yeast *MND1 *gene (At*MND1*). Loss of At*MND1 *did not affect normal vegetative development but caused fragmentation and missegregation of chromosomes in male and female meiosis, formation of inviable gametes, and sterility. Analysis of the *Atmnd1 Atspo11-1 *double mutant indicated that chromosome fragmentation in *Atmnd1 *was suppressed by loss of *Atspo11-1*. Fluorescence in situ hybridization (FISH) analysis showed that homologous pairing failed to occur and homologues remained apart throughout meiosis. At*MND1 *showed strong expression in meiocytes as revealed by RNA in situs.

**Conclusion:**

We conclude that At*MND1 *is required for homologous pairing and is likely to play a role in the repair of DNA double strand breaks during meiosis in *Arabidopsis*, thus showing conservation of function with that of *MND1 *during meiosis in yeast.

## Background

The formation of at least one crossover between pairs of homologous chromosomes is necessary for their correct segregation at meiosis I. The stages of interactions between homologous chromosomes that lead to crossover formation have been broadly grouped as: an initial localization of homologous chromosomes within the same region, mediated by interstitial interactions; close pairing and strand exchange at the DNA level as a part of recombination; and synapsis between homologous chromosomes together with completion of recombination [1].

Recombination at the DNA level in yeast and in other organisms is initiated by double strand breaks (DSBs) made by Spo11 [2,3]. Interaction between DSBs and a homologous intact chromosome can lead to crossover and noncrossover recombination products which are formed by two different pathways [4]. Processing of DSBs by 5' end resection yields 3' single-stranded ends that asymmetrically invade a homologous chromosome and lead to the formation of a double-Holliday junction intermediate which has been proposed to account for the majority of crossovers [5,6]. Interaction between homologous chromosomes at the sites of DSBs is promoted by the action of the RecA-like strand exchange proteins Rad51 and Dmc1 [7,8]. Several lines of evidence suggest that Rad51 and Dmc1 have different but overlapping functions [9,10] and interact with distinct sets of proteins in promoting recombination [11-13]. Rad51 acts in mitosis and in meiosis [14] whereas Dmc1 is meiosis specific [15].

*MND*1 was identified in *Saccharomyces cerevisiae *using three different screens based on genetic and functional genomic approaches that were directed at identifying genes that played a role in meiotic recombination and/or chromosome segregation [16-18]. The *mnd1 *mutant shows defects in nuclear division, meiotic recombination, and repair of DSBs. Mnd1 has been shown to act as a complex with Hop2 [18,19] and the Mnd1/Hop2 complex localizes to chromosomes independently of Rad51 and Dmc1 [18,20]. Genetic studies have provided evidence that Hop2 and Mnd1 act in the same pathway as Dmc1 and Rad51 [17-21]. Biochemical studies using yeast, human, and mouse orthologues have provided evidence that Mnd1/Hop2 stimulates the strand exchange activity of Dmc1 and that of Rad51 [19,22,23]. The interaction of Mnd1 with Hop2 has been shown to promote the interaction of Hop2 with Dmc1 and stimulate the strand exchange activity of Dmc1 [24]. Additional roles for Mnd1/Hop2 that have been proposed are in promoting interhomologue associations at DSBs through interaction with the axial elements or other proteins perhaps by relieving structural constraints [18,20,25] and in the designation of DSBs for noncrossover recombination [26].

Orthologues of *MND1 *have been identified in protists, fungi, plants, and animals and some of these have been characterized and shown to have closely related functions [27]. In yeast an *mnd1 *disruption has been reported to cause defects only in meiosis and does not result in sensitivity to radiation induced DNA damage [17]. However, an *Arabidopsis *mutant, *Atmnd1-Δ1 *has been recently shown to be sensitive to gamma radiation indicative of a role in mitotic repair, and also to undergo chromosome fragmentation during meiosis [28]. Here we have used the same mutant allele to analyze the role of the At*MND1 *gene in meiosis. We show that At*MND1 *is required for homologous pairing, an early step in the recombination process and that chromosome fragmentation in the *Atmnd1 *mutant is likely to be due to defective repair of meiotic DSBs. We also show that consistent with its role in meiosis, At*MND1 *is strongly expressed in meiocytes.

## Results

### At*MND1 *shows increased expression in reproductive tissues

**Figure 1:**
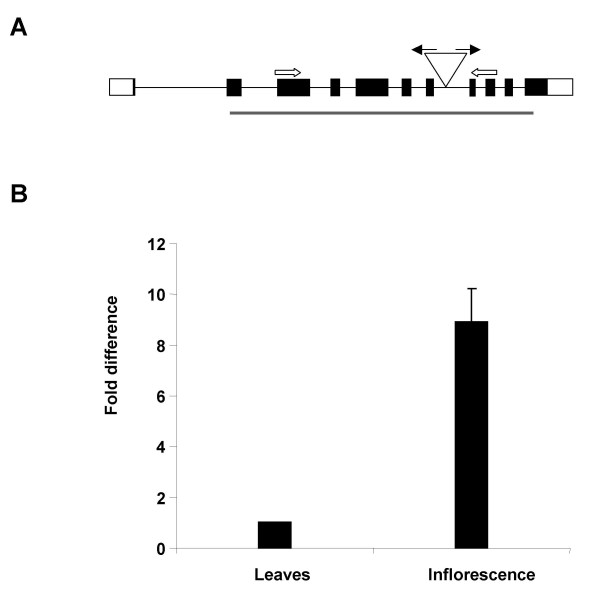
**Gene structure and expression of At*MND1***. (A) Line diagram showing the site of T-DNA insertion in the At*MND1 *gene. Exons and the UTR's are indicated as black boxes and clear boxes respectively. Inverted triangle in the seventh intron represents the site of T-DNA insertion. The black and white arrows represent the LB1 and the gene-specific primers used to genotype plants. The black bar below the gene represents the Mnd1 domain in the protein. (B) Real Time RT-PCR analysis of relative expression of At*MND*1 in somatic and reproductive tissues. The constitutive GAPC gene was used as the normalization control.

The *Arabidopsis *genome contains a single orthologue of *MND1 *(At*MND1*) corresponding to the annotated gene ID No. At4g29170 and supported by a cDNA (Accession No. AA063855). The encoded protein shows 26% identity (47% similarity) to Mnd1 and is 230 aa in length which is close to that of Mnd1 (219 aa). Expression of At*MND1 *was compared between rosette leaves and inflorescence using real time PCR (Fig. 1B). The results indicated a 9-fold higher expression of At*MND1 *in reproductive tissues over leaves consistent with a possible role in reproductive development.

### Mutation of At*MND1 *causes male and female sterility due to production of defective gametes

**Figure 2:**
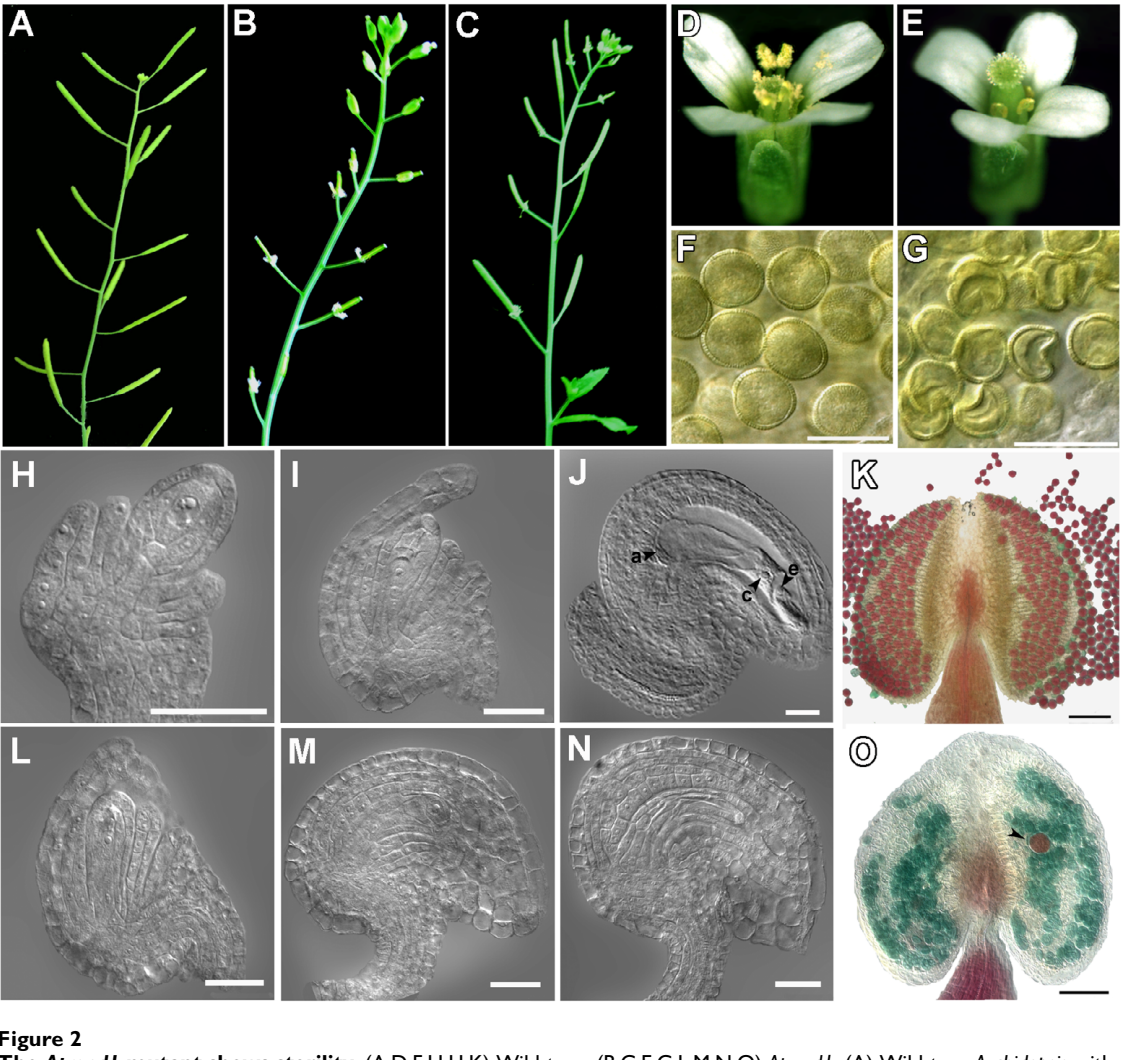
**The *Atmnd1 *mutant shows sterility**. (A,D,F,H,I,J,K) Wild-type, (B,C,E,G,L,M,N,O) *Atmnd1*. (A) Wild-type *Arabidopsis *with elongated siliques. (B) Sterile *Atmnd1 *plant with non-elongated siliques. (C) Mutant plant transformed with a wild-type At*MND1 *gene displaying restoration of fertility. (D) Wild-type flower. (E) Mutant flower showing under-developed stamen and stigma devoid of pollen grains. (F) Cleared anther containing round and uniformly sized pollen grains. (G) Mutant anther containing shriveled pollen grains. (H) Stage 2–4 ovule displaying the megaspore mother cell (MMC). (I,L) Stage 3-1 ovule showing the functional megaspore (J) Cleared wild type mature embryo sac; a, antipodals; c, central cell; e, egg cell. (K,O) Alexander staining for pollen viability; arrowhead indicates a rare purple viable pollen grain in the mutant. (M) Mature ovule with female gametophyte arrested at the uninucleate stage. (N) Mature ovule containing degenerating material instead of an embryo sac. Scale bars: K, O, 50 μm; H-J, L-N, 20 μm. Ovule stages are according to [54].

To examine the function of the At*MND1 *gene we obtained bulked T4 seeds of an insertion line SALK_110052 [29] carrying a T-DNA insertion in At*MND1 *and identified 2 plants that were homozygous for the insertion. Both plants were found to be sterile (Fig. 2) whereas 14/14 plants that were not homozygous for the insertion were fertile. No defects in vegetative development were observed. The progeny of a single plant that was heterozygous for the insertion segregated 84:23 fertile:sterile consistent with a single gene recessive trait (0.5 > p > 0.25 for a single gene model; 0.001 > p for a two gene model). 31 plants at random were genotyped with respect to the presence of the insertion and all 4 that were found to be homozygous for the insertion were also sterile indicating that the phenotype was closely linked to the insertion (0.001 > p for an unlinked gene model). The mutant showed a greater than 100-fold reduction in At*MND1 *expression suggesting that At*MND1 *function is severely reduced and is probably null (data not shown). To determine whether sterility was caused by mutation of At*MND1*, a 3.9 kb genomic fragment comprising the At*MND1 *gene and its promoter region was cloned into a plant binary vector pCAMBIA1300 [30] and transformed into *Arabidopsis *plants that were heterozygous for the *Atmnd1 *insertion allele, using in-planta transformation. 4 T1 transformant plants that were homozygous for the *Atmnd1 *insertion allele were identified out of 24 screened and all 4 were fertile, whereas among untransformed plants in a segregating population, 12 were identified to be homozygous for the *Atmnd1 *insertion allele and all were sterile (Fig. 2B) which demonstrates that the sterile phenotype is caused by mutation of At*MND1*.

Reciprocal crosses were carried out between wild type and mutant to identify the developmental defects underlying sterility, and the results in Table 1 indicate that the mutant shows both male and female sterility. Observation of anthers showed that the pollen grains in the mutant were shrunken (Fig. 2F,G) and inviable (Fig. 2K,O) as determined by Alexander staining [31]. Also the anther lobes remained below the level of the stigma as a result of reduced elongation of the anther filament. Likewise, examination of cleared ovules showed that in the mutant, postmeiotic development of the female gametophyte was arrested at the functional megaspore stage followed by degeneration of the functional megaspore (Fig. 2H–J,L–N; Table 2). About 0.6% of the developed ovules contained a mature embryo sac, which is consistent with the small number of seeds produced in the mutant. These results indicated that the mutant is male and female sterile due to both pollen and embryo sac formation being defective.

**Table 1:** Reciprocal crosses between wild type and *Atmnd1 *mutant.

Female parent	Male parent	No. of seeds per silique
*Atmnd1*	Wild type	1 +/- 1
Wild type	*Atmnd1*	0
Wild type	Wild type	58 +/- 5

Data shown are from a minimum of ten crosses.

**Table 2:** Female gametophytic defects in the *Atmnd1 *mutant.

Ovule Stages	1n		2n		4n		8n		MES		Degen	
	
	m	+	m	+	m	+	m	+	m	+	m	+
3-1	31	100	ND	ND	ND	ND	ND	ND	ND	ND	69	ND
3-2	25	60	9	40	ND	ND	ND	ND	ND	ND	66	ND
3-4	39	2	4	40	7	52	ND	5	ND	ND	50	0.8
3-6	32	ND	6	2	0.4	7	0.2	7	0.6	83	60	1.2

Stages of female gametophyte development relative to ovule stages in the *Atmnd1 *mutant and wild type. A total of 759 and 637 ovules were scored for the *Atmnd1 *mutant and wild-type respectively. Megagametogenesis defects are evident from stage 3-1 in case of the mutant, which leads to embryo sac arrest and eventually its degeneration. Ovule stages are according to [54]: 3-1, 3-2, and 3-4 are postmeiotic stages of the sporophyte with respect to elongation of the integuments where the wild type gametophyte would normally reach the 1n, 2n, and 4n stages; 3-6 corresponds to a mature ovule prior to fertilization. MES, Mature embryo sac; Degen, Degenerated embryo sac; M, mutant; +, wild type; ND, Not detected.

### At*MND1 *is strongly expressed at meiosis

**Figure 3:**
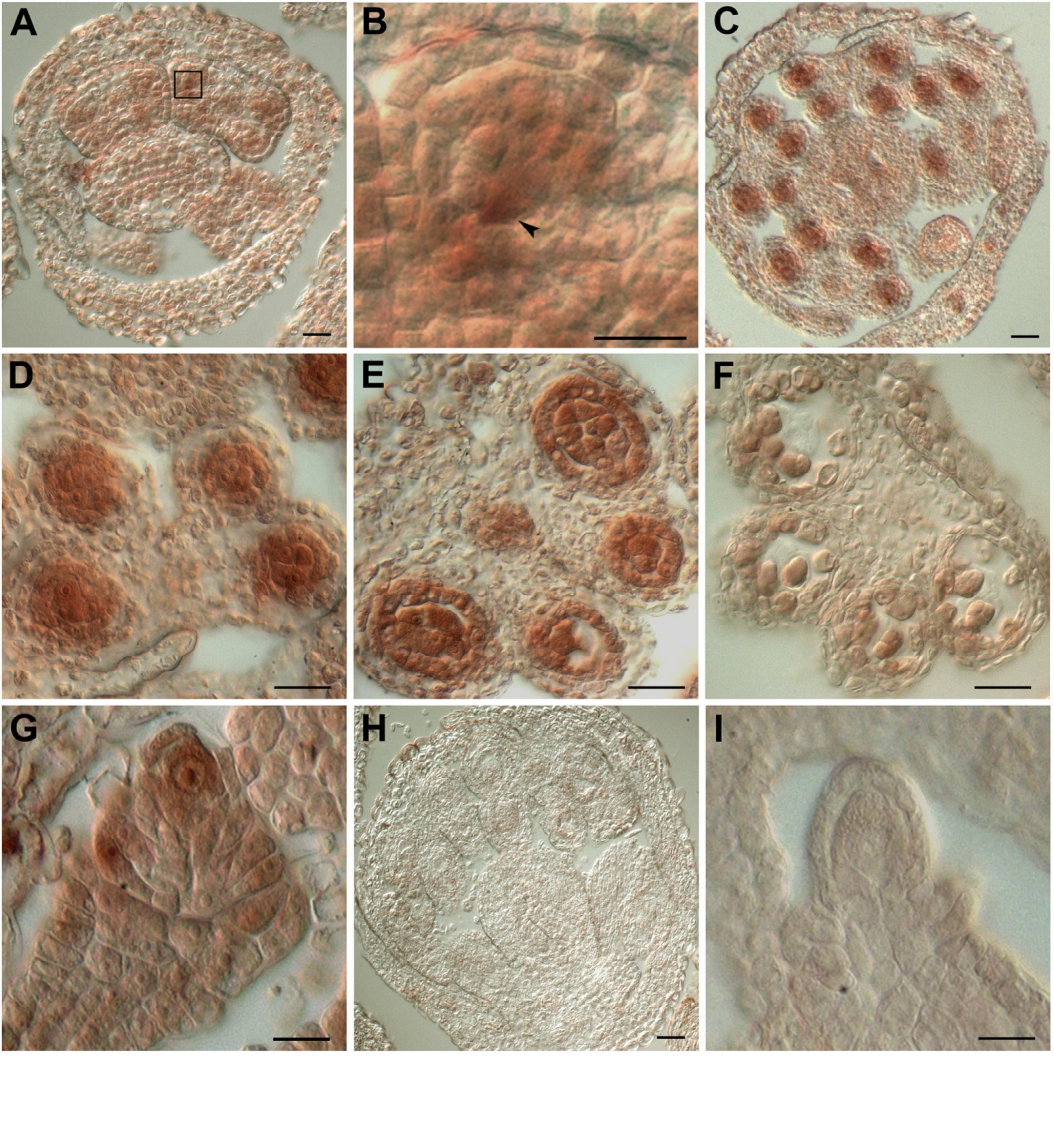
**Expression of At*MND1 *in male and female meiocytes**. RNA in-situ hybridization of At*MND1 *anti-sense RNA to flower buds. (A-F,H) Transverse sections of anthers. (G,I) Transverse sections of pistils. (A) Increased levels of expression first detected in stage 4 anther locules. (B) Magnification of inset in (A) showing increased expression in sporogenous cells (arrowhead). (C,D) Stage 5 anthers showing strong expression in pollen mother cells and tapetum. (E) Stage 6 anther containing meiotic cells. (F) Expression declines at the tetrad stage. (G) Increased expression in megaspore mother cell. (H,I) Sense controls. Scale bar: 25 μm.

To obtain more detailed information on the expression pattern of At*MND1 *in relation to the mutant phenotype we examined expression in inflorescence tissue sections by RNA in-situ hybridization using antisense RNA complementary to At*MND1 *cDNA (Fig. 3). A basal level of expression was observed throughout reproductive tissues. In addition strong expression of At*MND1 *was found within anther lobes at meiotic stages (Fig. 3A–E). The earliest increase in expression was detected in sporogenous cells at anther stage 4 [32]. Strong expression was observed in stage 5 anthers within microspore mother cells and also the tapetum. A high level of expression continued to be observed within meiotic cells during anther stage 6 and declined after meiosis. An increase in expression of At*MND1 *was also detected within the megaspore mother cell in ovules although not as strongly as observed in microsporocytes (Fig. 3G).

### At*MND1 *is required for homologous pairing

**Figure 4:**
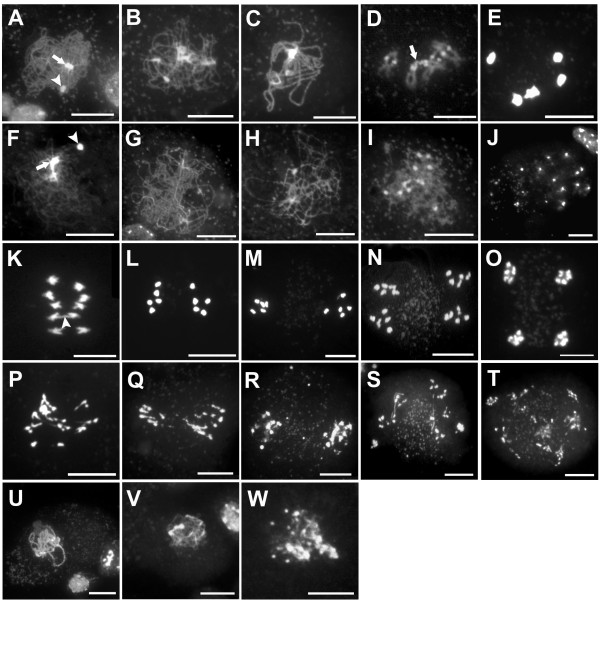
**Meiotic defects in the *Atmnd1 *mutant**. (A-E, K-O, U) Wild type, (F-J, P-T, V and W) *Atmnd1*. (A-T) Male meiosis, (U-W) female meiosis. (A,F) Unsynapsed elongated strands of chromosomes at late leptotene radiating from densely stained synizetic knot (arrow). The NOR is indicated by arrowhead. (B) Zygotene stage with partially synapsed chromosomes. (C) Synapsis is complete at pachytene and chromosomes have a shorter and thicker appearance. (D) Late diplotene stage, where bivalents have undergone partial decondensation of the arms but not at centromeric regions. Arrow indicates the NOR. (E) Diakinesis with brightly stained bivalents. (G) Unsynapsed chromosomes at stage corresponding to zygotene with the chromosomes remaining as univalents. (H) Pachytene equivalent stage showing irregular and unsynapsed univalent chromosomes. (I) Diplotene stage with patchy and fragmented chromosomes. (J) Occurrence of more than ten brightly stained spots indicates univalent chromosomes and their fragmented form at diakinesis. (K) Five separating univalents at early anaphase I. Residual chiasma can be seen in one of the separating bivalents (arrowhead). (L) Late anaphase I. (M) Metaphase II. (N) Anaphase II. (O) Telophase II. (P) Anaphase I with numerous chromosome fragments migrating to either pole. (Q) Abnormal segregation of fragmented chromosomes at late anaphase I. (R) Fragmented chromosomes aligned at the metaphase II plate. (S) Anaphase II, showing scattered chromosome fragments and bridges probably representing sister chromatid cross over. (T) Telophase II with polyads. (U) Female meiocytes at pachytene with fully synapsed chromosomes. (V) Corresponding stage as in U with chromosomes appearing fuzzy, remaining as univalents. (W) Female meiocyte at late prophase I showing extensive chromosome fragmentation. Scale Bars: 10 μm.

The expression pattern of At*MND1 *and the phenotype of the At*MND1 *mutant are together suggestive of a defect in meiosis. We therefore compared meiotic prophase stages from the mutant and wild type using spread preparations of meiotic chromosomes following the method of Ross et al., 1997 [33]. The initial stages of meiotic prophase corresponding to early leptotene were seen to occur in the *Atmnd1 *mutant and thread-like chromosomes were apparent (Fig. 4). Association of the nucleolar heterochromatin present on chromosomes 2 and 4 as well as the synizetic knot, which is formed during late leptotene concomitant with pairing and the start of synapsis [34] could also be observed (Fig. 4A,F). However, abnormalities could be detected starting at the zygotene stage, both with respect to the appearance of the chromosomes and the organization of pericentromeric heterochromatin. Chromosomes in the mutant appeared less compact when compared to wild type and pericentromeric heterochromatin regions were more extended and unpaired than at the corresponding stage in wild type (Fig. 4B,G). During the course of zygotene, the differences became more pronounced, and synapsis was defective. The thickening of chromosomes along segments of their length representing synapsed regions, that characteristically appears during zygotene and is complete by pachytene, did not take place in the mutant (Fig. 4C,H). At diplotene the chromosomes appeared as a diffuse and fragmented mass with 10–12 separate spots of condensed pericentromeric heterochromatin (Fig. 4D,I). At diakinesis, separated chromosomes and fragments could be clearly distinguished (Fig. 4E,J). The sorting of these fragments at anaphase I and II was irregular and bridges could be observed (Fig. 4K–N,P–S). Following meiosis polyads that contained a variable number of fragmented chromosomes were formed (Fig. 4O,T). These gave rise to defective spores that did not form viable pollen. Female meiosis in the mutant was also defective and chromosome fragmentation was observed (Fig. 4U–W). These data suggested the possibility that the mutant is defective in homologous pairing and synapsis and the accumulation of fragments may arise from defects in repair of DSBs.

**Figure 5:**
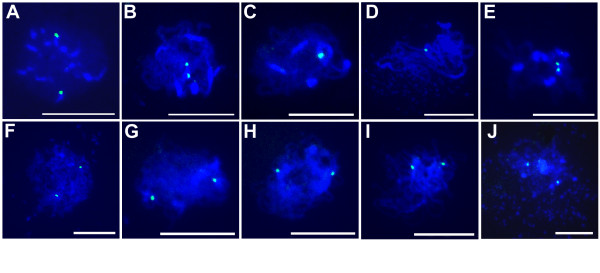
**FISH analysis of homologous pairing in wild-type and *Atmnd1 *plants**. (A-E) Wild type, (F-J) *Atmnd1*. (A,F) Early leptotene with two green signals indicating that homologous chromosomes are unpaired. (B,G) Two signals observed at leptotene-zygotene transition. (C) Late zygotene stage with only one signal indicating that the chromosomes have synapsed. (D) Pachytene stage with one signal. (E) Desynapsis occurring at centromeres during diplotene as evident by two closely appearing signals. (H) Late zygotene stage, with two signals placed far apart indicating unpaired state of homologous chromosomes. (I) Pachytene equivalent stage, where two signals are evident (J) Diplotene with two widely separated signals. Scale Bars: 10 μm.

To examine homologous pairing we carried out FISH experiments (Fig. 5) using a telomere repeat based oligonucleotide probe that hybridizes strongly to the centromere of chromosome 1 but not to centromeres of the remaining chromosomes [35]. In wild type, two well separated signals were observed at leptotene indicating that chromosomes were unpaired (Fig. 5A). A single signal was observed at late zygotene (46/46 nuclei) and pachytene (23/23 nuclei) indicative of pairing and synapsis having taken place (Fig. 5C,D). At diplotene, twin signals close to each other (6/6 nuclei) were seen indicative of centromere regions having desynapsed (Fig. 5E). These twin signals again merged and at metaphase I only a single signal was seen (data not shown). The *Atmnd1 *mutant showed two widely separated signals starting from leptotene and throughout all subsequent stages (58/61 nuclei for zygotene and 42/43 for pachytene) indicating that homologous pairing as well as synapsis was defective (Fig. 5F–J).

### Chromosome fragmentation in At*mnd1 *is suppressed by a mutation in At*SPO11-1*

**Figure 6:**
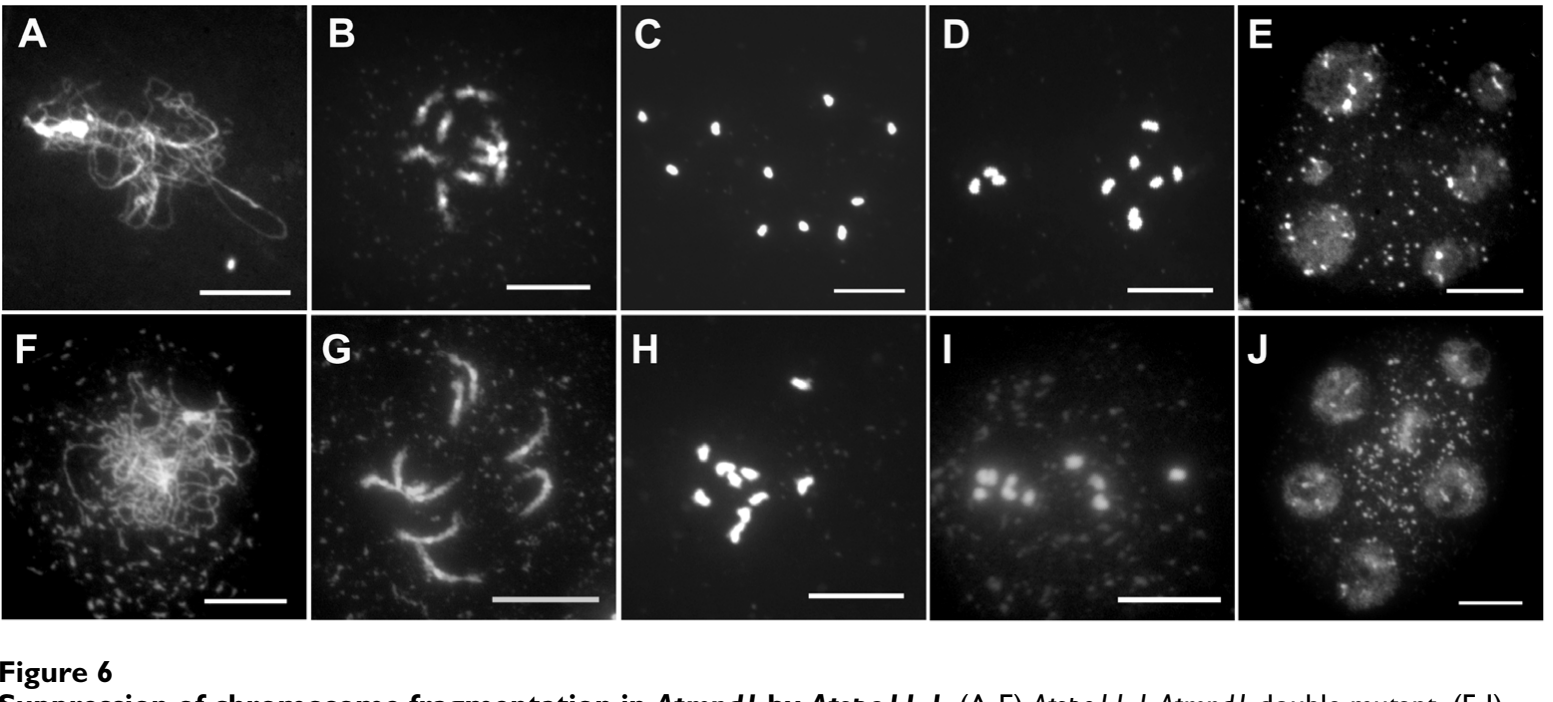
**Suppression of chromosome fragmentation in *Atmnd1 *by *Atspo11-1***. (A-E) *Atspo11-1 Atmnd1 *double mutant. (F-J) *Atspo11-1 *single mutant. (A,F) Pachytene equivalent stage with unpaired chromosomes. (B,G) Diplotene stage showing ten condensed univalents; four nucleolus organizing chromosomes are attached together at the NOR. (C,H) Diakinesis with ten brightly stained univalent chromosomes. (D,I) Anaphase I showing 7:3 and 6:4 unequal segregation of chromosomes respectively. (E,J) Polyad. Scale Bar: 10 μm.

The chromosome fragmentation phenotype together with the absence of homologous pairing suggested that the *Atmnd1 *mutant was defective in recombination possibly due to defects in the repair of meiotic DSBs. AtSPO11-1 is one of three SPO11 homologues in *Arabidopsis *and is specifically required for recombination and synapsis during meiosis [36,37]. To test whether fragmentation was dependent upon At*spo11-1*, a plant that was heterozygous for *Atmnd1 *was crossed to a SALK insertion line (SALK_146172) that was heterozygous for a T-DNA insertion in the seventh intron of At*spo11-1 *[29]. F1 plants that carried both insertions were identified in the F1 and homozygous *Atmnd1 Atspo11-1 *double mutants were obtained in the F2. Analysis of male meiotic chromosome spreads of the double mutant and comparison to the *Atspo11-1 *single mutant indicated that the chromosome fragmentation phenotype of *Atmnd1 *was suppressed by *Atspo11-1 *(Fig. 6). Chromosomes in the double mutant did not undergo fragmentation after diplotene (Fig. 6B,C) and remained as intact univalents that segregated randomly at the first meiotic division (Fig. 6C,D,H,I). The double mutant phenotype resembled that of the *Atspo11-1 *mutant. *Atspo11-1 *is therefore epistatic to *Atmnd1*.

## Discussion

Pairing and recombination between homologous chromosomes at meiosis relies on search for homology using resected ends that are created at the sites of DSBs. This search is mediated by the action of RecA-like strand exchange proteins Rad51 and Dmc1 which bind to single stranded DNA and promote the formation of joint molecules [7,8]. The strand exchange activity of Dmc1 and Rad51 is stimulated by Hop2 and Mnd1 which cooperate together as a complex [18,19]. Both Hop2 and Mnd1 are required in yeast for homologous pairing and meiotic DSB repair [16-18,20]. We have shown in this study that the *Arabidopsis *orthologue of *MND1*, At*MND1 *is required for homologous pairing during meiosis in *Arabidopsis *where it is likely to play a role in the repair of meiotic DSBs. At*MND1 *also shows strong expression in meiocytes.

The early defects in the *Atmnd1 *mutant with respect to overall appearance of chromosomes during meiosis were a lack of thickening during zygotene and absence of subsequent synapsis at pachytene. Fragmentation of chromosomes became apparent at diplotene and isolated univalents and fragments were first visible at diakinesis. FISH analysis using a centromere 1 specific probe indicated that homologous pairing did not take place in the mutant during zygotene and homologous chromosomes remained apart throughout meiotic prophase and meiosis I.

The meiotic phenotype of *Atmnd1 *is similar to that caused by a mutation in *AHP2 *which encodes the *Arabidopsis *orthologue of *HOP2 *[38]. In both cases there is chromosome fragmentation and a defect in homologous pairing. The failure to synapse and the appearance of fragmented chromosomes late in meiotic prophase I is a feature of several *Arabidopsis *mutants that are implicated in processing and repair of DSBs [39,40]. The observation that A*tspo11-1 *suppressed the chromosome fragmentation phenotype of *Atmnd1 *supports the interpretation that *Atmnd1 *is also defective in meiotic DSB repair. A major difference between the meiotic phenotype of yeast *mnd1 *and that for *Atmnd1 *in *Arabidopsis *is the absence of meiotic arrest in *Arabidopsis *whereas *mnd1 *shows prophase arrest which is alleviated by a mutation in *MEC1*, a major regulator of DNA damage induced checkpoints [25]. A lack of arrest is also seen in the case of *ahp2 *whereas *hop2 *shows prophase arrest [20]. The failure to arrest in the case of *Arabidopsis *is likely to be due to the absence or leakiness of meiotic DNA damage checkpoints and has also been observed for other *Arabidopsis *meiotic mutants for which the yeast counterparts show prophase arrest [41].

The yeast *MND1 *gene is expressed and functions only in meiosis and is not considered to play a role in mitotic DNA repair [17]. The At*MND1 *gene is dispensable for somatic development, however the *Atmnd1 *mutant is defective in mitotic DNA repair and At*MND1 *is induced in response to gamma irradiation [28] pointing to an evolutionary difference between yeast and plants with respect to the role of *MND1*. In plants the major pathway for repair of DSBs in somatic cells is non-homologous end joining (NHEJ) whereas in yeast the homologous recombination pathway predominates, which may explain the requirement for Hop2/Mnd1 in promoting efficient repair of DSBs and the maintenance of genome integrity in somatic cells [42]. Orthologues of *MND1 *and *HOP2 *are not present in *C. elegans *and *Drosophila melanogaster *both of which do not require DSBs for homologous synapsis at meiosis [43].

In addition to being strongly induced in meiocytes at the time of meiosis we found that At*MND1 *is also expressed in the tapetum at the same time. We have earlier noticed this to also be the case for the *DUET *gene which has a male meiosis specific phenotype [44]. It is possible that the tapetal expression at the same time as in meiocytes may reflect an overlap in the expression profile between tapetal cells and microspore mother cells which form adjacent layers and are both descended from the archesporial cell. Indeed the secondary parietal cells that are the precursors of the tapetum appear to retain the developmental potential to form meiocytes as revealed by mutations in *EXS/EMS1 *[45,46] and *TPD1 *[47] where microsporocytes are formed in place of tapetal cells. We also note that the onset of increased expression of At*MND1 *appears to be at anther stage 4 in a region occupied by sporogenous cells that are the precursors of male meiocytes. This stage is prior to the formation of meiocytes and initiation of recombination. These observations would suggest that the regulatory mechanisms responsible for increased expression of At*MND1 *in reproductive tissues may be distinct from those for DNA damage inducible expression [28].

## Conclusion

In summary we have shown that At*MND1 *is required for homologous pairing and repair of DSBs during meiosis in *Arabidopsis*. Loss of At*MND1 *does not affect normal vegetative development but causes male and female sterility due to fragmentation and defective segregation of chromosomes in meiosis.

## Methods

### Plant material and growth conditions

All the plants described in this study were *Arabidopsis *ecotype Col-0. The T-DNA insertion lines SALK_110052 and SALK_146172 used in this study were obtained from the Arabidopsis Biological Resources Centre, Ohio State University. Plants were grown as described previously [48].

### Characterization of the T-DNA Lines

Genomic DNA was extracted from the SALK T-DNA insertion lines using the method of Dellaporta et al.,1983 [49]. Presence of the T-DNA insert in *Atmnd1 *was confirmed by PCR using a left border outwardly directed primer (LB1) in combination with a gene-specific primer (AtMND1F1) flanking the site of insertion. Based on our analysis, there are at least two tandemly placed T-DNA inserts placed next to each other such that the orientation in the genome is LB-RB-LB. Junction fragments on either ends of T-DNA were amplified using primer combinations AtMND1F1 and LB1 and AtMND1R1 and LB2. Sequencing of the AtMND1R1-LB2 product revealed the presence of a 86 bp deletion within intron 7. Primers AtMND1F1 and AtMND1R1 were utilized to amplify the wild type allele. For the *Atspo11-1 *line the presence of the T-DNA insert was confirmed using the primer TSPO11R in combination with LB1 and wild type copy using TSPO11F in combination with TSPO11R. Homozygous insertion lines showed a phenotype that was the same as that of Atspo11-1-1 [36].

### Complementation analysis

A full-length genomic clone spanning 3891 bp (AGI coordinates 14382003–14385894) was amplified using primers MndF1 and MndR1 that incorporates restriction sites, *Bam*H1 at 5' and *Eco*R1 at 3' end respectively. The amplification was done using TripleMaster PCR system (Eppendorf) as per the manufacturer's instructions. The resulting 3.89 kb fragment was cloned into the pGEM-T vector (Promega) followed by sequencing of the fragment. Restriction digestion with enzymes *Bam*H1 and *Eco*R1 was performed to release the fragment, which was sub-cloned into the binary vector pCAMBIA1300. The fragment was mobilized into *Agrobacterium *strain AGL1 by tri-parental mating. The *in planta *transformation was carried out on heterozygous *Atmnd1 *plants by vacuum infiltration as reported earlier [50]. The transformants were selected on medium comprising of 1% bacto agar, 1% sucrose, 1 mM KNO3 and 50 μg/ml of hygromycin B and genotyped by PCR using Atmnd_out_FG_Rev1 and Atmnd_out_FG_For1 primers.

### Expression analysis by Real Time RT-PCR

Total RNA was isolated using Trizol (Sigma) as per the manufacturers protocol. cDNA was synthesized from 5–7 μg of RNA using the Superscript first strand synthesis system (Invitrogen) with Oligo dT primers. Real Time PCR reactions were done in a 10 μl volume comprising of primer, cDNA template and 1× SYBER Green PCR master mix (Applied Biosystems). GAPC was used as the internal normalization control. PCR was performed on the ABI Prism 7900 HT Sequence Detection System (Applied Biosystems) in a 384 well reaction plate according to the manufacturer's recommendations. Primers were MNDRTF1 and MNDRTR1 for At*MND1 *and GAPRTF1 and GAPRTR2 for *GAPC*. Cycling parameters consisted of 2 minutes incubation at 50°C, 10 minutes at 95°C and 40 cycles of 95°C for 15 seconds, 57°C for 30 seconds and 67°C for 30 seconds. Each PCR reaction was performed in triplicate and the experiment were repeated twice. Specificity of the amplifications was verified at the end of each PCR run using ABI prism dissociation curve analysis software. Results from the ABI Prism 7900 HT Sequence Detection System were analyzed further using Microsoft Excel. Quantification of mRNA was calculated from threshold points (Ct values) located in the log-linear range of real time PCR amplification plots.

### RNA In situ Hybridizations

In situ hybridizations were carried out as described earlier [48]. Anti-sense RNA specific to the At*MND1 *gene was used as probe along with sense control. We used the full-length At*MND1 *cDNA amplified from the cDNA clone using *Nco*1F and *Eco*R1R primers and subcloned into pGEM-T vector for strand specific probe synthesis. Floral stages are according to [51].

### Microscopy

Developmental analysis of whole mount anthers and ovules was done after fixing and clearing the inflorescence in methyl benzoate as described previously [46]. The slides were observed on a Zeiss Axioplan 2 Imaging microscope under DIC optics using a 40× oil immersion objective. Pollen viability was examined using the method of Alexander staining [31]. Meiotic chromosome spreads were prepared, analyzed, and staged based on chromosomal morphology and with respect to the stage of the surrounding tapetal cells, according to Ross et al., 1996 [33] with minor modifications as described in [52]. Chromosomes were stained with DAPI (1 μg/ml) and observed on a Zeiss Axioplan 2 Imaging microscope using a 365 nm excitation and 420 nm long pass emission filter and a 100× oil objective. Images were captured on an Axioplan CCD camera using Axiovision (version 3.2) and processed using Adobe Photoshop 6.0.

### Fluorescence In situ Hybridization (FISH)

Meiotic spreads were carried out as described above and FISH analysis was done according to [53] with incorporation of minor modifications. The hybridization mix was prepared with 5' FITC labeled probe FITC-(CCCTAAA)_6 _[35] at a concentration of 5 μg/ml in 50% deionised formamide, 2× SSC and 10% dextran sulphate. The hybridization mix was denatured on a hot block for 3 minutes at 100°C and immediately cooled on ice. The slides were denatured separately with 100 μl denaturation mix comprising of 70% deionised formamide, 2× SSC and 50 mM sodium phosphate buffer pH 7.0 mounted under a 24 × 50 mm^2 ^cover slip and incubated at 80°C for 5 minutes. After the incubation, the slides were washed in ice-cold 70% ethanol for two minutes followed by dehydration in 70%, 90% and 100% ethanol respectively (2 minutes each) and air dried. Denatured probe (100 μl) was then applied to the slides and covered with a 24 × 50 mm^2 ^cover slip. Hybridization was carried out in a moist chamber for 18 hours at 37°C. Post-hybridization washes were performed in 2× SSC, pH 7.0 (two washes each for 5 minutes at room temperature) followed by 2× SSC for 5 minutes at 42°C. Chromosomes were counter stained with DAPI (1 μg/ml) in Vectashield (Vector Laboratories). Fluorescence detection was done on a Zeiss Axioplan 2 Imaging microscope equipped with epifluorescence illumination and distinct filters for DAPI and FITC using a 100× oil immersion objective. The images were captured with a Axioplan CCD camera using Axiovision software (Zeiss, version 3.2) and processed using Adobe Photoshop 6.0.

### Primers used in this study

1. AtMND1F1 ACCGAAGAAGGGTGTAATTAGTCAGTC

2. AtMND1R1 ATTGTCGCAGTGTGAAGATGTTATCTG

3. MndF1 CAGGAGAATTCAAACCGAGAACATGAAACAGATCC

4. MndR1 GACGAGGATCCAATCATAGAAACAGACTTGGACC

5. Atmnd_out_FG_Rev1 CCTGGACCAGAAGAAGGTAAGGGTTTTG

6. Atmnd_out_FG_For1 GAGCTATTCACATGCTTAACAAGTTGCTAACAG

7. Nco1 F GCTCGCCCATGGCTATGTCTAAGAAACGGGGAC

8. EcoR1 R GCGGAGAATTCCTAAGCTTCATCTTGTACTAGCT

9. LB1 AACCAGCGTGGACCGCTTGCTGCAACTC

10. LB2 CAGGGCCAGGCGGTGAAGG

11. MNDRTF1 TCGATGATGATCTTGTTGCGAA

12. MNDRTR1 TCACACTGATCAACAAGTTCTGCt

13. GAPRTR2 CAGTCTTCTGAGTAGCAGTGATTGA

14. GAPRTF1 AGCACGAATACAAGTCCGACCT

15. TSPO11R ACTGTGATAACAATGCAGCGGTTCG

16. TSPO11F CAGCACAATCCATTGTGGACCGTGC

## Authors' contributions

AP and IS conceived and designed the experiments. AP did the basic genetic and phenotypic analysis of plants together with BN, MM, VS, and VV. TR characterized the *Atspo11-1 *mutant allele and BN generated the *Atmnd1 Atspo11-1 *double mutant. MR did the meiotic chromosome analysis. MR and AP did the FISH analysis. JS did the Real Time PCR and RNA in situ expression analysis of At*MND1*. IS wrote and BN helped edit the manuscript. All coauthors reviewed and approved the final manuscript.

## Note added in proof

While this manuscript was under review, a paper was published by Kerzendorfer et al., also describing work on the role of At*MND1 *in homologous pairing and recombination [55].
